# Cell Pattern in Adult Human Corneal Endothelium

**DOI:** 10.1371/journal.pone.0019483

**Published:** 2011-05-13

**Authors:** Carlos H. Wörner, Alicia Olguín, José L. Ruíz-García, Nuria Garzón-Jiménez

**Affiliations:** 1 Instituto de Física, Pontificia Universidad Católica de Valparaíso, Valparaíso, Chile; 2 Escuela Universitaria de Óptica, Universidad Complutense de Madrid, Madrid, España; 3 Instituto de Oftalmología Avanzada, Madrid, España; University of Reading, United Kingdom

## Abstract

A review of the current data on the cell density of normal adult human endothelial cells was carried out in order to establish some common parameters appearing in the different considered populations. From the analysis of cell growth patterns, it is inferred that the cell aging rate is similar for each of the different considered populations. Also, the morphology, the cell distribution and the tendency to hexagonallity are studied. The results are consistent with the hypothesis that this phenomenon is analogous with cell behavior in other structures such as dry foams and grains in polycrystalline materials. Therefore, its driving force may be controlled by the surface tension and the mobility of the boundaries.

## Introduction

Biological cell patterns are a contemporary theme of study, both for its intrinsic biological interest and also by their potential medical applications. Current research has been recently summarized [1] and lately, it is possible also to mention the works on the hexagonal packing of Drosophila wings [2] the results of on Drosophila retina [3], and a theoretical approach on geometric order in Drosophila imaginal discs [4].

Concerning the main theme of the present note, a concise treatment of the biology of the corneal endothelium appears in Bourne's review [5]. It is a well established fact that corneal endothelial cells form a monolayer of mosaic-like cells, with two-dimensional tessellation on the posterior surface of the cornea. Also, it is well-known that its cellular morphological characteristics evolve with aging: some cells grow and others disappear. Polymegethism (cell size) and polymorphism (geometric cell parameters) are usually measured by optical microscopy. Several eye anomalies can be detected by a physical examination of this tissue, and therefore its “normal” characteristics need to be studied thoroughly.

The purpose of this note is to analyze the results of the increase in cell size in adult humans (or equivalently, the reduction in cell density) reported in the literature. It is important to note that results taken from different populations in diverse regions of the world are used, and also that the “normal adult eye” differs for these distinct populations depending on their geographical location. There is ample evidence for this last fact, as described, for example, by some recent papers [6], [7]; this would therefore imply that the endothelial cell characteristics also differ from place to place.

Being so diverse the sample population of the ten analyzed countries, our concern is to examine the rate of growth of the cell size. Therefore, it is the main proposal of this note to establish that the rate of increasing in the mean cell size shows similar behavior in all considered populations. In addition, it is suggested that this aging behavior is governed by capillary driving forces (surface tension) acting on the cell boundary.

With this goal in mind, we will examine evidence from other systems that present cell growth. Surface tension driven cellular patterns (by short, “foam-like” systems) have been studied in the current literature associated with grain growth in polycrystalline materials and cellular development in foams. Although there is not complete agreement in the way these complex systems evolve, recently, a theoretical treatment for two-dimensional grain growth in a stochastic framework has been proposed [8]. Also, there are other proposed theoretical models [9]. An appropriate vision of the “state of the art” on this matter appears in some seminal reviews [10]-[11]. On the other side, the Physics of foams have been comprehensively revised in a recent book [12] and reviewed in current works appearing in the literature [13], [14]. As well, there have been approaches that simulate the grain growth behavior in polycrystalline materials by using soap froth patterns [15], [16].

## Results

Endothelial cell densities have been measured in adult humans by several research groups. There are results on the North American and Japanese populations [17]; Japanese data [18], Italian results [19], Iranian population [20], Pakistani data [21], Indian reports [22], and Phillipines [23]. Also, it exists earlier results from American population [24], Danish records [25]. Recently, Chinese data [26] and Portuguese measurements [27], [28] had been published.

It is important to emphasize that the present note only refers to normal adult eyes. Although other cases are not considered, it is useful to state that there also exists substantial knowledge on the parameters of this cellular pattern for other types of population, i.e. children [29], animals [30] and also for abnormal endothelia [31]. Obviously, the aforementioned references do not cover all the literature with reference to the important issue concerning non-adult human eyes.

In the following paragraphs, we analyze the evidence that support our proposal of a “foam-like” behavior for the endothelial cell pattern.

**Figure 1:**
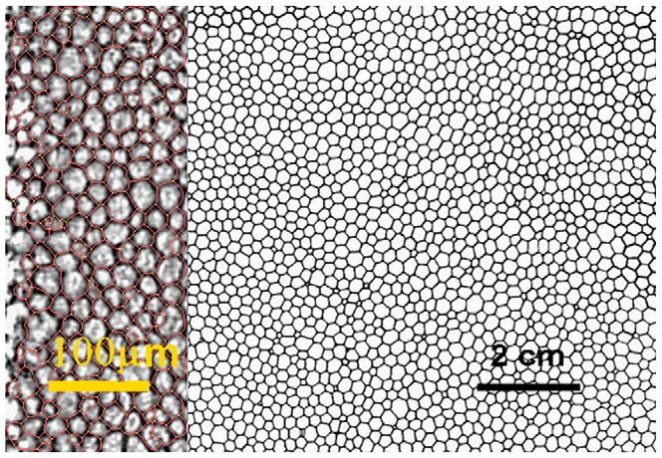
The image on the left shows typical endothelial cells. On right, a two-dimensional foam is shown. Note the similitude of the outlines and the difference in scale.

1. Morphology. There is a striking similitude between the endothelium cell pattern and other cellular, non-biological patterns appearing in different contexts. All these configurations also show aging, i.e. its properties vary according the elapsed time. Fig. 1 shows a typical endothelium pattern and a soap froth pattern. Both are two-dimensional tessellations and although the scales are different, the distribution of the cells appears to be very similar. Analogous patterns also appear in other scenarios, i.e. lipids, metals, ceramics, Potts model, iron garnet, cucumber, geography and cosmological models as remarked in the literature [13]. In all these phenomena, the driving force for the growth of the cells is the interface properties of the boundaries (also called capillary effects), and their movement is governed by the surface tension and the mobility of these boundaries.

2. Hexagonallity. Further evidence of this similitude is the tendency to hexagonallity. In these patterns, cells tend to stabilize in a hexagonal structure, as stipulated by the so called Mullins-von Neumann law for two-dimensional area growth of a single cell [32], [33]. This law states that a cell with more than six sides grows and a cell with less than six sides shrinks, with equilibrium being obtained by hexagonal cells, in accordance with the equation:
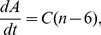
(1)


In this equation, *dA/dt* is the growth rate of a cell with *n* sides and *C* is a constant involving a geometrical factor and the surface tension and mobility of the boundary. This law is in agreement with the prevalence of hexagonal morphology in endothelial cell patterns, a well documented fact in the current literature [34]. It is to be stressed that the Mullins-von Neumann law is valid for individual cells, whereas measured growth kinetics usually refers to mean values (see Eq. 2).

3. Kinetics of growth. One of the more known experimental facts about the kinetics of these phenomena is the increase in mean cell size (or the corresponding decrease in cell density). This behavior appears also neatly in the analysis of soap froth domain growth [13] and grain growth in polycrystalline materials [10], [11], [35].

**Figure 2:**
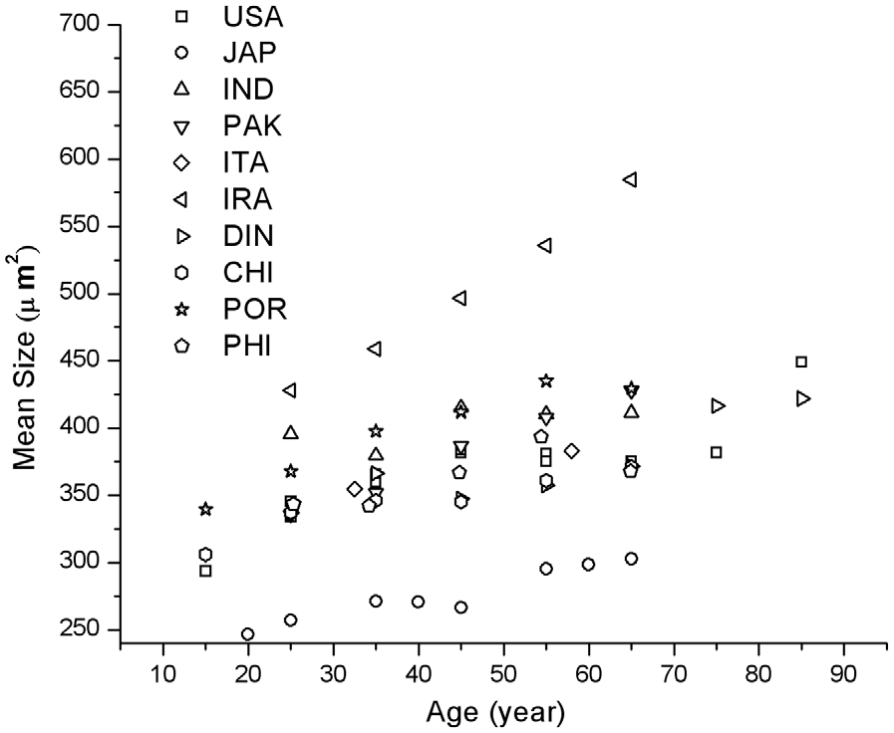
Mean size kinetics reported in the literature.

In order to further explore this analogous behavior, we will analyze the numerical values measured of this parameter. The mean cell size kinetics in aging -taken from the corresponding values reported in the current literature- is depicted in Fig. 2. (In some examples from current literature aging kinetics are reported in terms of cell density. In these cases, the data has been homogenized and represented in terms of the corresponding cell mean size.).

The data for the considered reports show a strong tendency to have a constant slope. They appear in a band bounded by the measured Iranian values at the upper side and the Japanese values at the bottom. Data from India, Pakistan, Italy, Denmark, China, Philippines and Portugal and both records from the USA may be grouped as a set of similar results. As previously mentioned, different behavior depending on geographical origin is to be expected.

**Figure 3:**
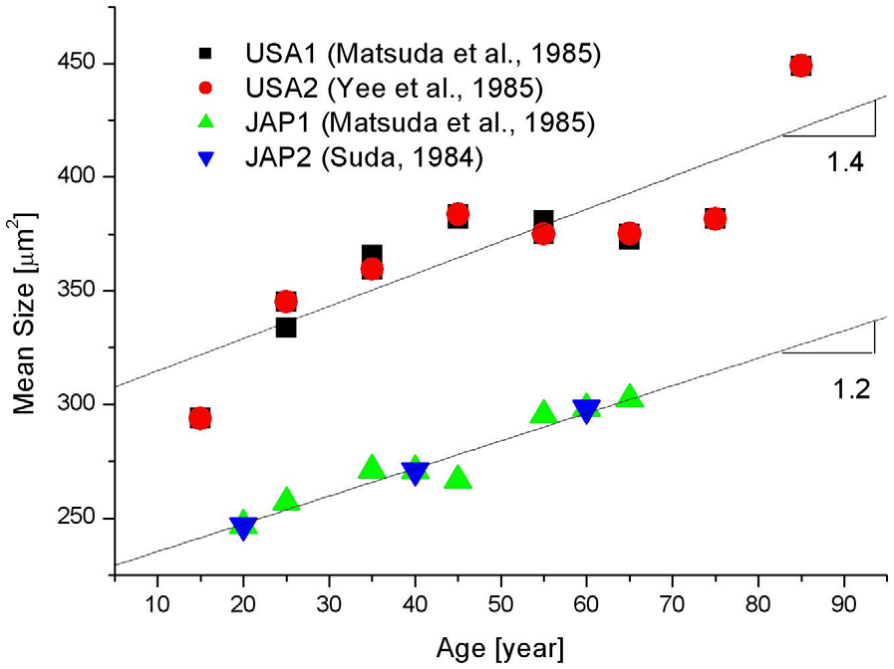
Mean size kinetics corresponding to USA and Japan adults. Slopes for both cases are indicated in the figure.

As already mentioned, the data show dispersion. To better expose our argument, we plot in Fig. 3 the results corresponding to USA and Japan measured values. In each of these cases the data are obtained from two different independent works; USA data [17], [24] and Japan data [17], [18]. It is observed that a linear fit for each of these groups shows similar slope (1.2 µm^2^/year and 1.4 µm^2^/year, respectively) so supporting our assertion that each set of data show a linear behavior. There is not sufficient evidence to assert that all considered data fits with the same slope and only an estimate of the order of 1 µm^2^/year, may be stated.

This incremental behavior confirms a well established qualitative fact on the aging kinetics of endothelial cells, but it is necessary to stress that this result, for adult populations, shows proportionality with time (that is, the displayed experimental points show a common slope) This outcome is true irrespective of the choice of population included in the different research samples.

In other terms, the considered patterns follow the “parabolic law” (it scales with size squared). This fact is a well established result [10]–[12], [14] on the kinetics of this type of patterns, and states that the mean area grows linearly with time, according to the equation,
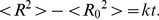
(2)


In this equation, *R* is the cell size, *<R^2^>* is a measure of the mean cell area at time *t* and *<R_0_^2^>* is the initial mean cell area. The *k*-value represents the slope of the corresponding plot and therefore can be inferred from the displayed data.

4. Statistical distribution. There is not agreement of the shape of the cell size distribution for foam-like structures. As already noted [14], “…determining the distribution of surfaces and volumes while the foam coarsens remain a subject of active research”. In fact, only to mention a recent intent on this matter it is possible to study a just published paper and references therein [36]. However, as show in the above mentioned literature, there is an ample accord in the statement that this distribution is scale-invariant.

**Figure 4:**
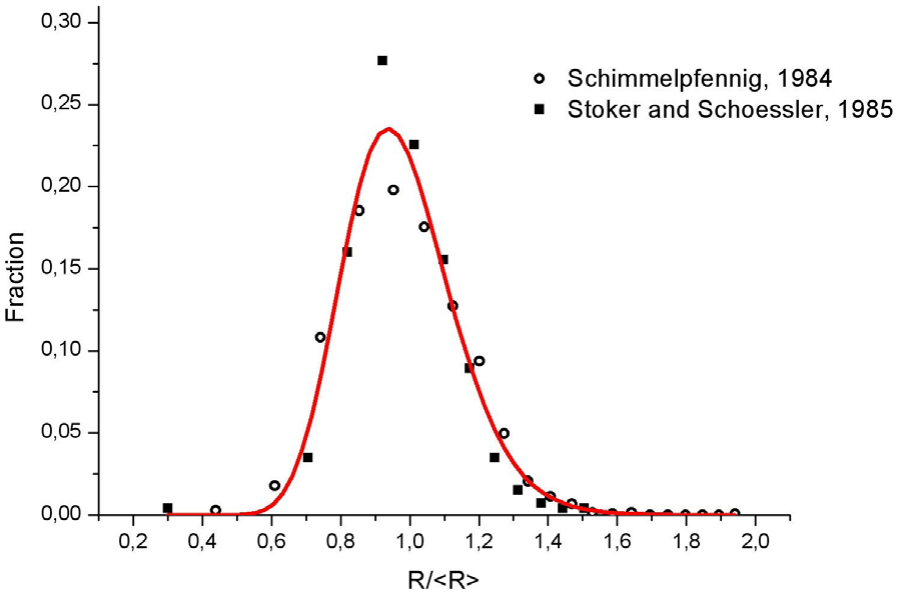
Cell fraction distribution. The experimental points correspond to an analysis of Schimmelpfennig and Stoker and Schoessler's data. Also shown is the log-normal fitting.

In absence of a definitive answer to the question of the distribution function and for the purposes of this note we will use the log-normal distribution. There are solid evidence on this assumption in the corresponding literature [36]–[39]. With this assumption in mind, by using the photographic record already published [34] and data extracted from an existing paper [40], the cell distribution at the central endothelial zone was examined. These statistics were calculated from the 1516 cells appearing in a plate of Schimmelpfennig's paper and 1500 cases considered in the Stoker and Schoessler's work. Consequently, we depict in Fig. 4 a log-normal plot of the data, which shows an excellent fit with the experimental result. (r^2^ = 0.97; χ^2^/dof = 0.0002; note the positive skewness).

## Discussion

In summary, we have reviewed the data concerning entothelian cell growth. What's more, it is here proposed that the aging of cells in endothelial tissue follows the laws of the cellular two-dimensional patterns already studied in other structures, as suggested by its morphological structure, the linearity of the mean cell growth rate and the prevalence of cells with a hexagonal shape. Strong added evidence is given by the correlation shown in the analysis of the scaled statistical cell distribution. In other words, corneal epithelial cells form a foam-like system, restricted to the experimental evidence found in human adults.

The fitting of the data to linear forms is a consequence of the original already published data. The use of an exponential fitting -as suggested in some references- is a valid hypothesis only if we include infant patients. Data in humans [26], [29], [41], [42] and mice [30] show experimental results. The rapid fall in cell density for children is clearly related with the growth of the cornea itself, that is, the pattern of cells has a more spacious growing environment. In the adult case, the size of the cornea is fixed and the cells have a limited space to grow. When we discard the data corresponding to infants, the linear relationship is recovered.

Perhaps, a comment on the evidence of different patterns in central and peripheral corneal zones seems in order. As already noted [34], [43], the shapes and distribution of the cells from these different zones give dissimilar (although not strongly different) morphological results. In the framework of the analogy with other patterns, it is possible to speculate that this effect is due to the boundary constraints caused by the finite size of the cornea. In other words, as cells grow, stagnation effects occur which are more significant in the border area. This braking effect has been well documented for thermal grooves in thin films [44] and in domain growth of soap froths and polycrystalline materials with quenched impurities [15], [16]. Therefore, it is possible to assume that cell growth in the central endothelium corneal zone is analogous to “free” cell growth without constrains.

It is worth noting that these conclusions put a sound basis for a clever insight of Schoessler and Ornsborn [45]. Referring to the aging of growth pattern, they wrote: “we believe…that a more likely explanation is that many cells are “shrinking” and others are expanding to fill in the space.”

As a final remark, it is a well know fact that endothelian cells do not replicate in-vivo (at least in the necessary amount to replace disappearing cells). Instead, they do reproduce in-vitro [46]. The crucial point seems to be, why the reproductive behavior of these cells is different in an aggregate than in a single cell? We guess that this paper may offer a new perspective on this question.

## Methods

As noted in the introduction section, data for cell densities in adult human endothelial cells were obtained from the literature [17]–[28]. Data for cell size fraction distribution (Fig. 4) were obtained from the works of Schimmelpfenning [34] and Stoker and Schoessler [40]. These numerical results were processed by standard graphical and statistical software.
